# Alcohol Consumption and Obesity: An Update

**DOI:** 10.1007/s13679-014-0129-4

**Published:** 2015-01-08

**Authors:** Gregory Traversy, Jean-Philippe Chaput

**Affiliations:** Healthy Active Living and Obesity Research Group, Children’s Hospital of Eastern Ontario Research Institute, 401 Smyth Road, Ottawa, ON K1H 8L1 Canada

**Keywords:** Alcohol intake, Wine, Beer, Liquor, Energy balance, Appetite, Body weight, Adiposity

## Abstract

Recreational alcohol intake is a widespread activity globally and alcohol energy (7 kcal/g) can be a contributing factor to weight gain if not compensated for. Given that both excessive alcohol intake and obesity are of public health interest, the present paper provides an update on the association between alcohol consumption and body weight. In general, recent prospective studies show that light-to-moderate alcohol intake is not associated with adiposity gain while heavy drinking is more consistently related to weight gain. Experimental evidence is also mixed and suggests that moderate intake of alcohol does not lead to weight gain over short follow-up periods. However, many factors can explain the conflicting findings and a better characterization of individuals more likely to gain weight as a result of alcohol consumption is needed. In particular, individuals who frequently drink moderate amounts of alcohol may enjoy a healthier lifestyle in general that may protect them from weight gain. In conclusion, despite the important limitations of current studies, it is reasonable to say that alcohol intake may be a risk factor for obesity in some individuals, likely based on a multitude of factors, some of which are discussed herein.

## Introduction

Alcoholic beverages have been consumed by humans since prehistoric times for a variety of reasons. Nowadays, recreational alcohol intake is common across the globe and health and social problems resulting from alcohol consumption are becoming a concern [1]. Although moderate alcohol use is recommended, excessive alcohol consumption is the third leading cause of premature death in the United States (behind smoking and obesity) [2]. Among the many problems associated with heavy alcohol drinking, the relationship between alcohol intake and body weight has been extensively studied over the past years [3•, 4, 5].

Based on the fact that 1 gram of alcohol provides 7.1 kcal (29 kJ) and studies showing that energy consumed as alcohol is additive to that from other dietary sources [5], increased energy intake with alcohol use can certainly promote a positive energy balance and ultimately weight gain. However, a clear cause-and-effect association between alcohol intake and weight gain is not apparent based on the mixed and conflicting available evidence on the topic. Given that both excessive alcohol intake and obesity are of public health concern, a better understanding of the association between alcohol consumption and excess body weight is warranted.

Therefore, the objective of this article is to provide an update on the link between alcohol intake and obesity. Furthermore, factors that may explain the conflicting findings in this research area are discussed. Finally, recommendations for future research are provided to promote a better understanding of the possible obesity-promoting effects of energy intake from alcohol.

## Alcohol Intake and Obesity: Observational Evidence

Observational studies on the effect of alcohol intake on obesity date back almost 30 years [6]. It has been examined across small and large cohorts, in many countries, across various ethnicities and age groups [4]. Within the large body of observational research, contradictory findings exist, which warrant further exploration [3•, 4].

### Cross-sectional Evidence

Among cross-sectional studies, a common trend appears to be that alcohol intake is not associated with body mass index (BMI) in men, while either negatively or not associated with BMI in women [4]. Indeed, several cohorts ranging from 10,482 to 138,031 individuals have shown no correlation (or a small negative correlation) between alcohol intake and BMI in men, and a small negative association with BMI in women [6–13]. Other studies have found that alcohol intake is positively correlated with BMI in men or in both sexes; however, an analysis of recent studies suggests that this may be due to differences in intake patterns [4, 7]. For instance, several studies in adults have found that the amount or intensity of drinking per drinking occasion is positively correlated with BMI, while the frequency of drinking is negatively correlated, suggesting that frequent light drinking might offer a protective effect [14–16]. Furthermore, several studies have found that only excessive or heavy drinking is correlated with increased measures of adiposity. Wannamethee, Shaper & Whincup [17] found that in men, drinking <20 drinks per week was not associated with higher BMI, waist circumference (WC) or waist-to-hip ratio (WHR) compared to non-drinkers. These anthropometric measures were much higher than non-drinkers in men who consumed >21 drinks per week [17]. Similarly, Coulson et al. [18] found higher BMI, percent body fat, and waist circumference in individuals drinking five or more drinks per day compared to non-drinkers. Other studies have shown J-shaped curves when comparing BMI, WC, and WHR among male and female drinkers, with light drinking being negatively associated with adiposity indicators compared to heavy drinking, or abstention [19–23].

To test the proposal that patterns of alcohol intake may be an important influence on overweight and obesity, several recent studies have examined the effects of binge drinking. Most recently, Sheldon and Knott [24] recorded the energy intake from alcohol on the days that individuals had their highest intake. The risk of obesity was 70% higher in the heaviest drinking group compared to the lightest. These individuals had obtained ≥ 75% of their total daily energy intake from alcohol compared to the lightest group which had < 24% of their daily energy intake from alcohol on their heaviest drinking day [24]. Binge drinking has been associated with greater risk of obesity and large WC in other studies [19, 25]. Also, Fan et al. [26] had individuals retrospectively estimate their age at peak alcohol intake and earlier reported peak alcohol intake was associated with more heavy drinking episodes and abdominal obesity. However, the retrospective nature of this study creates the potential for inaccuracies or biases in participants’ memory of their drinking behaviors.

Despite the recent body of cross-sectional evidence suggesting the benign or potentially protective effect of frequent light drinking on body weight and obesity, several studies have found conflicting results. Sung et al. [27] found a very modest positive linear relationship between alcohol intake and BMI in men. However, the BMI of the highest drinking group varied only by 0.4 points compared to non-drinkers, although the trend was statistically significant. In overweight participants this relationship was even less pronounced (a difference of 0.2 points) [27]. Alcacera et al. [28] found that in older participants (>39.2 years old), alcohol intake was positively correlated with BMI, but no association was seen in younger participants. Vagstrand et al. [29] found that alcohol intake was positively associated with body fat in adolescent girls, but negatively associated with body fat in adolescent boys [29]. Finally, Croezen et al. [20] found a positive association between alcohol intake and risk of overweight in 13–14 year olds. Among 15–16 year olds, however, they found a J-shape relationship, with only the heaviest drinkers having a higher risk of overweight [20]. This suggests that there may be differences in alcohol metabolism between age groups; however, it is important to note that this study assessed only the amount of alcohol per drinking occasion, and not drinking frequency patterns.

Overall, the majority of cross-sectional studies since 2005 have demonstrated that frequent light to moderate alcohol intake does not seem to be associated with obesity risk. Heavy drinking and binge drinking, however, are more likely to carry such an association with excess body weight [16, 19, 22–25]. Alcohol intake may also promote overweight and higher body fat percentage in adolescents or older adults [20, 28, 29]. These studies are limited in their ability to demonstrate cause-and-effect relationships or changes in weight status over time. Thus, a thorough review of recent prospective studies is also important.

### Longitudinal Evidence

Prospective studies have looked at the association between alcohol intake and adiposity gain in various populations, with follow-up periods ranging from several months to 20 years [4, 30, 31]. Results of these studies have been varied and do not provide a clear picture. Several studies have found no association or a negative association between alcohol intake and changes in weight, BMI or other measures of adiposity [12, 30, 32–39]. Other studies have found such an association only in women, while finding a positive association between obesity risk and alcohol intake in men [40]. This study, however, did not specify the amount of alcohol intake, and did not control for participant’s physical activity (PA) levels [40]. Conversely, one study found no association between alcohol intake and increases in WC in men, but a small positive association in women [41]. There have also been recent studies that have found a general positive association between alcohol intake and weight gain [42]. This study, however, did not control for PA levels and only assessed alcohol intake on a yes/no scale [42].

Changes in alcohol intake patterns have been associated with weight gain in prospective studies [15, 31, 43]. French et al. [15] found that men who increased their frequency or amount of alcohol intake over the follow-up period were more likely to experience increases in BMI and WC. They noted, however, that the differences were relatively modest [15]. Mozafarrian et al. [31] also found that changes in drinking behavior were associated with increases in weight. Over four years each increase of one drink per day was associated with a predicted increase of approximately 0.19 kg [31]. This relationship was strongest for beer, suggesting a possible influence for the type of alcohol ingested [31].

Finally, other recent studies have shown that heavy drinking may be more of a risk factor for weight gain than light-to-moderate drinking [44•, 45–47]. MacInnis et al. [44•] found that lower BMI and WC were seen after 12 years of follow up in light and moderate drinkers than heavy drinkers or abstainers. Sayon-Orea et al. [46] found that drinking more than seven times per week was associated with increased risk of weight gain and development of overweight and obesity. Shütze et al. [47] found that male light-to-moderate beer drinkers had smaller increases in WC and weight than non-drinkers or heavy drinkers over 8.5 years of follow up. However, conversely, in women they found a dose–response relationship between beer intake and weight gain and WC gain [47]. There are many reasons as to why the relationship between alcohol and adiposity varies between men and women, involving genetic and lifestyle factors, some of which will be discussed in depth below.

As with cross-sectional studies, the way by which alcohol intake is measured and categorized likely influences the interpretation of the results. Several studies have grouped all levels of individual alcohol intake above 30g/day as ‘heavy’ drinkers [12, 38]. Conversely, other studies examined alcohol intake more thoroughly, considering frequency and amount per drinking day separately [15]. French et al. [15] measured alcohol frequency ranging from 1–2 times per year to every day, while estimating the number of drinks per drinking day from 1–36. Such an analysis allows for a more complete description of participants’ drinking patterns, and is important as cross-sectional studies suggest that drinking frequency and intensity influence weight differently [14–16].

Collectively, the most recent prospective studies suggest that light-to-moderate alcohol intake is not associated with weight gain or changes in WC [44•]. Heavy drinking, however, has been more consistently associated with weight gain [45–47]. Furthermore, increases in alcohol intake patterns appear to promote weight gain [15, 31, 43]. Binge drinking behavior was not specifically examined in any of the recent prospective studies analyzed.

## Alcohol Intake and Obesity: Experimental Evidence

Several experimental studies have been conducted to examine the short-term effect of alcohol intake on feeding behavior and appetite control [3•, 5]. A recent review summarized a number of these studies, showing that alcohol ingested before a meal has frequently been shown to have a neutral effect on intake, or to increase intake, despite the added energy that come from the alcohol preload [5]. In these studies, alcohol appears to have no effect on appetite, or to increase appetite [5]. However, to date there have been few intervention studies conducted to experimentally examine the effects of regular alcohol intake on weight gain or obesity in humans. All of the available studies have examined moderate intake of alcohol, and the majority have reported results on beer and wine intake, but not other forms of alcohol [3•, 5]. Crouse and Grundy [48] looked at the effect of adding 630 kcal/day of alcohol to the diets of 12 men in a metabolic unit. There were no significant changes in weight for normal weight participants over the four-week intervention study. They however noted that about half of the obese participants gained weight, with the largest weight gain being 1.8 kg [48]. In a randomized crossover study, Cordain et al. [49] found that drinking two glasses of red wine (270 mL) with dinner daily for six weeks did not lead to changes in weight or body fat percentage in 14 men. They noted that self-reported nutrient intake and physical activity did not differ between conditions, although there may have been dietary compensation that was not accurately reported by their 3-day food logs [49]. Similarly, Cordain et al. [50] found that 10 weeks of wine intake equal to 6-7% of total energy intake (135 mL, five times per week) did not result in any significant change in body weight or fat percentage in 20 sedentary, overweight women. Also, Beulens et al. [51] reported similar results in 34 male adults with large WC, consuming 450 mL of red wine per day for 4 weeks, compared to consuming alcohol-free wine for the same time period. The effect of beer intake was examined by Romeo et al. [52] who found that one month of daily beer consumption (equivalent to 12g/day of alcohol for women and 24 g/day for men) did not result in significant increases in BMI or WC compared to abstention. Biceps skin fold was the only anthropometric measurement that was increased in their participants after the beer drinking condition [52]. Fletchner-Mors et al. [53] found that replacing 10% of total daily energy intake during a weight-loss intervention with either grape juice or white wine resulted in similar weight loss, with the white wine group showing a slightly higher (although not statistically significant) weight loss. In this case both diets were isoenergetic so this is not a surprising result, as the thermic effect of food was likely higher for white wine than grape juice [53, 54]. Finally, more recently, Cresci et al. [55] found that self-reported alcohol intake was not a significant predictor of success or failure in losing 5% of body weight during a 6-month weight loss intervention.

Overall, the available experimental evidence reviewed in this article suggests that moderate intake of alcohol does not lead to weight gain. The systematic review by Bendsen et al. [3•] suggests that this trend is less likely in experimental studies examining beer consumption exclusively. Also, the intervention periods in the aforementioned studies ranged from 4–10 weeks, and therefore may not have been long enough to identify the slight changes in weight that can accumulate over time to result in overweight or obesity. A modest increase in weight of one kilogram over a 10 week period seems insignificant but over five years this could result in up to 26 kg of weight gain if no compensation takes place. To our knowledge, there does not appear to be any experimental evidence specifically testing the effects of heavy/binge drinking, or of drinking spirits or a combination of alcohol sources on weight gain/obesity. A summary of the studies examined in this article, organized by the trend between alcohol and weight gain/obesity can be found in Table 1.

**Table 1:** Summary of trends in cross-sectional, longitudinal, and experimental studies examining the link between alcohol intake and measures of adiposity

	Trend	Study (measurement)	
		Men	Women
Cross-sectional studies	No association	Colditz et al. [8] (BMI) Gruchow et al. [6] (BMI) Williamson et al. [13] (BMI) Croezen et al. [20] (In 15–16 year olds, overweight risk)	Alcacera et al. [28] (BMI) Croezen et al. [20] (In 15–16 year olds, overweight risk)
	Positive association	Alcacera et al. [28] (Only men above median age, BMI) Croezen et al. [20] (Only in 13–14 year olds, overweight risk) Sung et al. [27] (BMI)	
	Negative association	Liangpunsakul [9] (BMI, WHR) Rohrer et al. [10] (Obesity rate)	Colditz et al. [8] (BMI) Gruchow et al. [6] (BMI) Liangpunsakul [9] (BMI, WHR) Rohrer et al. [10] (Obesity rate) Skrzypczak et al. [11] (BMI, WHR) Williamson et al. [13] (BMI)
	J-shaped	Arif & Rohrer [19] (Obesity risk) Duvigneaud et al. [21] (combined WC and BMI) Lukasiewicz et al. [22] (WHR, BMI) Wakabayashi [23] (WC) Wannamethee, Shaper & Whincup [17] (BMI, WC, WHR)	Arif & Rohrer [19] (Obesity risk) Duvigneaud et al. [21] (combined WC and BMI) Tolstrup et al. [16] (BMI, WC) Wakabayashi [23] (WC)
	Binge/ heavy drinking positively associated	Arif & Rohrer [19] (Obesity rate) Coulson et al. [18] (BMI, WC, body fat %) Lee [25] (WC) Shelton & Knott [24] (Risk of obesity)	Arif & Rohrer [19] (Obesity rate) Lee [25] (WC) Shelton & Knott [24] (Risk of obesity)
	Frequency negatively associated, Intensity positively associated	Breslow & Smothers [14] (BMI) French et al. [15] (BMI) Tolstrup et al. [16] (BMI, WC)	French et al. [15] (BMI)
Longitudinal studies	No association	Arabshahi [32] (Change in BMI, WC) alkjaer et al. [33] (Change in WC) Holloway et al. [30] (Change in BMI) Pajari et al. [35] (Change in BMI) Romaguera et al. [41] (Change in WC) Tolstrup et al. [38] (Change in WC)	Arabshahi [32] (Change in BMI, WC) Economos et al. [43] (Weight gain) French et al. [15] (Change in BMI) Halkjaer et al. [33] (Change in WC) Holloway et al. [30] (Change in BMI) Pajari et al. [35] (Change in BMI) Sammel et al. [36] (Weight gain of >10lbs)
	Drinkers gain more over follow-up	Bell, Ge & Popkins [42] (Risk of weight gain) Hou et al. [40] (Risk of weight gain)	Bell, Ge & Popkins [42] (Risk of weight gain) Romaguera et al. [41] (Change in WC) Wanamethee et al. [14] (Only in African American participants, change in weight >5kg)
	Drinkers gain less over follow-up	Liu et al. [34] (Risk of major weight gain)	Hou et al. [40] (Risk of weight gain) Liu et al. [34] (Risk of major weight gain) Thompson et al. [37] (Change in weight) Tolstrup et al. [38] (Change in WC) Wang et al. [39] (Weight gain)
	Increases in drinking pattern positively associated	Economos et al. [43] (Weight gain) French et al. [14] (Change in BMI) Mozafarrian et al. [31] (Weight gain)	Wanamethee et al. [12] (Change in weight >5kg) Schutze et al. [47] (Change in WC)
	Positive association only in heavy drinking	Halkjaer et al. [33] (Wine only, change in WC) MacInnis et al. [44•] (Change in BMI, WC) Rissanen et al. [45] (Risk of weight gain) Sayon-Orea et al. [46] (Risk of weight gain, obesity) Schutze et al. [47] (Change in weight, WC)	MacInnis et al. [44•] (Change in BMI, WC) Rissanen et al. [45] (Risk of weight gain) Sayon-Orea et al. [46] (Risk of weight gain, obesity) Wanamethee et al. [12] (Change in weight >5kg)
Experimental studies	No significant effect of alcohol intake	Cordain et al. [49] (Weight, body fat %, others) Beulens et al. [51] (Weight, Body fat distribution) Fletchner-Mors et al. [53] (Change in weight) Crouse & Grundy [48] (Normal weight participants, change in weight) Romeo et al. [52] (Weight gain, WC)	Cordain et al. [50] (Body weight, body fat %) Fletchner-Mors et al. [53] (Change in weight) Romeo et al. [52] (Weight gain, WC)
	Positive impact of alcohol consumption	Crouse & Grundy [48] (Half of obese patients, change in weight) Romeo et al. [52] (Biceps skinfold only)	Romeo et al. [52] (Biceps skinfold)

BMI = body mass index, WC = waist circumference, WHR = waist-to-hip ratio

## Alcohol Intake and Obesity: Potential Mechanisms

In summarizing the recent literature it appears that light-to-moderate alcohol intake is less likely to be a risk factor for obesity than heavy drinking. Heavy drinking and binge drinking have been more consistently linked with adiposity. There are several lines of evidence suggesting the potential for alcohol to promote weight gain, and the contradictory results often seen in the literature have led to the development of alternative hypotheses regarding the influence of alcohol on body weight.

A review by Yeomans [5] highlights some of the potential explanations for alcohol’s influence on weight gain or obesity. First, as previously mentioned, energy from alcohol appears to be additive to energy from other sources [5]. Several studies suggest that consuming alcohol before or during a meal does not influence the amount of food eaten in that meal, despite increasing the energy density of the meal [5]. Thus, individuals do not appear to compensate for the added energy from alcohol in the short-term, and alcohol appears to have little effect on satiety [5].

Beyond adding energy to a meal, alcohol may actually stimulate food intake [5]. Of the 17 studies reviewed by Yeomans, ten showed increased food intake following alcohol consumption [5]. One explanation is that there is a learned association between alcohol and eating; however, several experimenters disguised the presence of alcohol in their protocols and still found increased energy intake [5]. It is unclear whether alcohol promotes food intake in the absence of hunger; however, it has been noted that alcohol may amplify individuals’ perception of appetite in response to food stimuli [5].

Alcohol has also been shown to influence a number of hormones linked to satiety. The results of several studies propose that alcohol may influence energy intake by inhibiting the effects of leptin, or glucagon-like peptide-1 (GLP-1) [56, 57]. To date, the evidence suggests that alcohol does not appear to increase appetite through the action of peptide YY (PYY), ghrelin, gastric inhibitory peptide (GIP), or cholecystokinin (CCK) [57–61]. Calissendorf et al. [58] found that alcohol did not increase plasma levels of neuropeptide Y (NPY); however, animal models have shown that central NPY levels are increased following alcohol consumption [62].

Alcohol can also influence hunger via several central mechanisms. The effects of alcohol on opioid, serotonergic, and GABAergic pathways in the brain all suggest the potential to increase appetite [62–65]. Given the complexity of the interplay between central and peripheral signals of satiety, more research needs to be performed in order to elucidate the precise biochemical mechanism driving food intake following alcohol consumption. A summary of the effects of alcohol on important appetite hormones and central neurological pathways in humans can be found in Table 2.

**Table 2:** Effect of alcohol on various peripheral hormones and central neurotransmitter systems related to hunger and energy intake

	Hormone/ neurotransmitter	Effect on hunger/ energy intake	Effect of alcohol intake on hormone/ neurotransmitter response	Reference(s)
Peripheral signals	CCK	Suppresses	Increase	Hajnal, Flores and Valenzuela [60]; Manabe et al. [61]
	Leptin	Suppresses	Decrease	Rojdmark, Calissendorff and Brisman [56]
	GLP-1	Suppresses	Decrease	Raben et al. [57]
	GIP	Suppresses	No effect	Raben et al. [57]
	PYY	Suppresses	No effect	Calissendorf et al. [59]
	NPY	Stimulates (is inhibited by leptin)	No effect	Calissendorf et al. [58]
	Ghrelin	Stimulates	Decrease	Calissendorf et al. [58]
Central neurotransmitter systems	GABA	Stimulates	Agonist	Koob [64]
	Opioids	Stimulates	Increase	Yeomans and Gra [63]; Widdowson and Holman [65]
	Serotonin	Suppresses	Decreases	Yeomans et al. [62]

CCK = cholecystokinin, GLP-1 = glucagon-like peptide-1, GIP = gastric inhibitory peptide, PYY = peptide YY, NPY = neuropeptide Y, GABA = gamma-aminobutyric acid

Aside from the immediate influence on appetite that comes from alcohol consumption, there are also effects on energy storage. Alcohol inhibits fat oxidation, suggesting that frequent alcohol consumption could lead to fat sparing, and thus higher body fat in the long term [62]. However, the results of the various cross-sectional and longitudinal studies examined in this review do not unequivocally support such a hypothesis. Finally, there is also evidence to suggest that traits that predispose individuals to binge drinking may also predispose to binge eating [66].

Although there is evidence to suggest that frequent alcohol intake may predispose to weight gain or obesity over the long-term, this effect is not strongly reflected in the recent research. This has prompted the development of several hypotheses. First, it has been found that alcohol intake increases energy expenditure, likely due in part to the fact that it has a high thermogenic effect [53]. It has also been suggested that some of the energy ingested as alcohol is ‘wasted’, due to the activation of the inefficient hepatic microsomal ethanol-oxidizing system (MEOS). The MEOS is induced through chronic alcohol intake, and the level of induction increases with increased intake [54, 67]. Oxidation of alcohol via the MEOS produces less ATP than oxidation via alcohol dehydrogenase, using the energy from alcohol intake primarily to enhance heat production [37, 54]. The extent to which wasted energy from regular alcohol consumption contributes to weight gain prevention is unclear.

It has also been suggested that individuals who frequently drink moderate amounts of alcohol may enjoy a moderate lifestyle in which exercise and food intake are modulated over the long term to accommodate for alcohol intake [15]. However, studies using food logs and self-reported physical activity levels have still shown a null or negative association between moderate alcohol intake and weight gain after controlling for these and other confounders, although they may fail to truly capture the habits of participants [4, 49]. While cross-sectional and longitudinal studies have controlled for a number of important lifestyle factors, there are many to consider when examining body weight regulation. It is highly likely that the paradoxical results seen in studies examining the effect of alcohol on weight gain and obesity are also the product of a multitude of factors beyond the individual’s ingestion habits. Future research must consider the other important factors that may influence the link between alcohol and obesity, some of which are discussed below.

## Factors that may Explain the Conflicting Findings between Alcohol Intake and body Weight

The mixed evidence with respect to alcohol’s role in promoting obesity is a product of many factors [e.g., gender, type, frequency and amount of alcohol consumed, drinking pattern (e.g., binge drinking), physical activity level, sleeping habits, depression symptoms, psychosocial problems, chronic illness, medication use, disinhibition eating behavior trait, history of alcohol use, predisposition to gain weight, etc.]. Not taking into account some of these potential confounding factors can certainly lead to biased estimates of the relationship between alcohol intake and body weight given that large inter-individual variations exist. Some of these confounding factors are further discussed below.

The association between alcohol intake and body weight is generally stronger in men than women [15], especially because of the amount and type of alcohol consumed by men. Alcohol has been reported to account for 16% of adult drinkers’ total energy intake in the United States [68], with men consuming about three times the amount consumed by women [68]. Men are also more likely to drink beer, which is carbohydrate rich, and provides more energy than wine per standard drink [5]. Consistent with this observation are the findings from a recent systematic review showing positive associations between beer consumption and measures of abdominal adiposity (also known as “beer belly”) in men; however, inconsistent results were found in women [3•].

Another important confounding factor to be considered is physical activity level. Many epidemiologic studies fail to consider lifestyle choices such as physical activity and sedentary behaviors despite the fact that increased energy expenditure may counter increases in energy intake through alcohol consumption [17, 23, 30, 32, 40, 42]. Furthermore, beer and spirit drinkers appear to have poorer dietary habits in general than wine drinkers [3•]. Thus, accounting for both sides of the energy balance equation (intake, expenditure and lifestyle habits) is crucial to evaluate adequately the association between alcohol intake and obesity.

Insufficient sleep has also been shown to be associated with greater alcohol consumption and excess body weight in adults [69, 70]. Specifically, sleeping less than 6 hours per night in adults is associated with greater alcohol intake, an increased risk of exceeding the guidelines for sensible weekly alcohol intake, and higher BMI. Furthermore, we showed that the combination of short sleep duration with disinhibited eating behavior is associated with greater alcohol intake and excess weight [69, 71]. These results emphasize the need to identify high-risk individuals (e.g., disinhibited short sleepers) who are in greater need of preventive strategies.

Genetic aspects can also play a role in the predisposition of individuals to gain weight as a result of alcohol consumption. Recent results showed that genetic polymorphisms affect susceptibility to alcoholism and may affect body weight via gene-associated differences in fuel utilization [72]. The authors found that alcohol dehydrogenase-1B (ADH1B) genotype (rs1229984) is a strong determinant of body weight in alcoholics. The more rapid ethanol elimination associated with the ADH1B*2 allele may result in less efficient utilization of ethanol as an energy source [72]. Those findings thus suggest that more research is needed to unravel genetic aspects involved in the connection between alcohol intake and weight gain.

Overall, obesity is a multi-factorial condition and it is difficult to truly assess the independent influence of alcohol intake on obesity risk. The observational evidence is hampered by the possibility of residual confounding by unmeasured variables and the experimental evidence is limited by the short-term follow-up period and the difficulty to control for all lifestyle habits under free-living conditions. The slow development of obesity and multi-faceted nature of this condition really complicates the possibility to show a cause-and-effect association between alcohol consumption and weight gain. Thus, we need to rely on short-term intervention studies and epidemiologic studies, each of which has clear limitations in showing an effect of alcohol intake on the vulnerability to gain weight. However, the preponderance of the evidence taken as a whole suggests that alcohol may be a risk factor for obesity in some individuals, especially when consumed in large quantities.

## Conclusions

Alcohol consumption has probably contributed to the excess energy intake associated with weight gain in some individuals over the past years. However, the available evidence is conflicting and hampered by important limitations that preclude a strong conclusion on the effect of alcohol intake on obesity risk. Moderation in drinking is still an important recommendation, together with a healthy lifestyle not conducive to weight gain.
